# Towards an integrated and proactive management of pulmonary hypertension in systemic sclerosis: a practical approach for early diagnosis and optimal patient management

**DOI:** 10.1093/rap/rkag077

**Published:** 2026-07-09

**Authors:** Dilia Giuggioli, Francesco Del Galdo, Michele D’Alto, Marco Matucci-Cerinic

**Affiliations:** Rheumatology Unit, University Hospital of Modena, Department of Medical and Surgical Sciences for Children and Adults, University of Modena and Reggio Emilia, Modena, Italy; Leeds Institute of Rheumatic and Musculoskeletal Medicine, LIRMM, Leeds, UK; NIHR Leeds Biomedical Research Centre, Leeds Teaching Hospitals Trust, Leeds, UK; Cardiology, Ospedali dei Colli–C.T.O. Hospital, Naples, Italy; Unit of Immunology, Rheumatology, Allergy and Rare Diseases (UniRAR), IRCCS San Raffaele Scientific Institute, Milan, Italy; Università Vita Salute San Raffaele, Milan, Italy; Inflammation Fibrosis and Ageing Initiative (INFLAGE), Division of Genetics and Cell Biology, IRCCS San Raffaele Scientific Institute, Milan, Italy

**Keywords:** systemic sclerosis (SSc), pulmonary arterial hypertension (PAH), diagnosis, care pathways, multidisciplinary team

## Abstract

Pulmonary arterial hypertension (PAH) is a severe vascular complication of SSc and a leading cause of disease-related mortality. Despite the availability of validated screening tools and treatment recommendations, diagnosis delay and suboptimal therapeutic implementation remain frequent in real-world practice. The 2022 European Society of Cardiology/European Respiratory Society guidelines and 2025 EULAR recommendations advocate systematic annual screening and initial combination therapy with an endothelin receptor antagonist and a phosphodiesterase type 5 inhibitor at PAH diagnosis. In SSc patients already receiving a dual combination, escalation to triple therapy including selexipag, or in selected cases switching to riociguat, should be promptly considered. Given the rapid progression and poorer prognosis of SSc-PAH, follow-up within 3 months of diagnosis is critical. Structured referral networks, implementation of the DETECT algorithm and involvement of a dedicated case manager and/or nurse can further improve timely diagnosis and continuity of care. Optimizing SSc-PAH management requires a proactive, integrated approach that bridges rheumatology and cardiology expertise.

Key messages

- SSc-associated PAH remains a leading cause of mortality, requiring systematic screening and early diagnosis.
- Implementation of validated screening algorithms and structured referral pathways enables timely identification and specialist management.
- SSc-associated PAH management should be rheumatologist-led to ensure continuity of care.

## Introduction

SSc is a systemic autoimmune rheumatic disease characterized by immune dysregulation, vasculopathy and skin and internal organ fibrosis [1, 2]. In SSc, a severe vasculopathy may lead to pulmonary arterial hypertension (PAH), occurring in ≈8–12% of patients and contributing to poor prognosis [1, 3]. Indeed, it is well known that SSc-associated PAH is an independent predictor of early death, with survival rates at 5 years of <60% [3, 4]. The progression of pulmonary vascular resistance and the onset of right ventricular dysfunction is often subclinical given the reserve capacity of both organs, leading to a diagnostic delay that is associated with a worse outcome [5]. Therefore, early identification is essential to provide the best therapeutic approach to improve survival rates. In 2022, European Society of Cardiology (ESC)/European Respiratory Society (ERS) guidelines recommended routine screening for PAH for all SSc patients during the first assessment and then on an annual basis [5, 6], while the EULAR recommendations for the treatment of SSc provided indications for appropriate therapy [7].

Despite clear guidelines, the dissemination of both screening and therapy recommendations is suboptimal to date. In routine clinical practice, systematic screening and specific diagnostic algorithms still need to be implemented, particularly for rheumatologists who are not familiar with PAH and lack access to multidisciplinary teams specializing in the management of PAH. Herein, we present a practical guidance to support rheumatologists in the screening and diagnosis of PAH in SSc, to promote timely referral to expert centres, facilitating proactive follow-up, with additional focus on therapy to ultimately improve patient outcomes.

## Diagnosis and referral of the SSc patient at risk for PAH: current gaps and limitations *vs* opportunities and strengths to implement current practice

### Gaps and limitations

Several gaps limit optimal care for SSc patients, with a diagnostic delay of ≈4 years between the onset of PAH symptoms and diagnosis [8]. Limitations in PAH early recognition and management are particularly marked in smaller or peripheral centres. As a result, underdiagnosis of PAH in SSc patients remains substantial, with real-world detection rates falling below the 8–12% prevalence reported in the literature [1] and estimated to be ≈6.4% in recent meta-analyses from observational reports [9].

In many non-specialist settings, the systematic screening of SSc remains inadequate due to lack of awareness of PAH. When echocardiography is performed, it is often limited to left heart assessment, without adequate evaluation of right-sided parameters. Cardiologists performing echocardiographic evaluation in SSc patients may not be sufficiently informed of the risk of PAH and the SSc-PAH haemodynamic complexity is a significant diagnostic challenge, as the phenotype may vary and may be difficult to distinguish without the support of specialized expertise [6]. Indeed, SSc patients may evolve over time from isolated post-capillary pulmonary hypertension (PH) related to left-heart diastolic dysfunction to combined pre- and post-capillary PH as pulmonary vascular disease progresses [2, 6]. For this reason, accurate classification requires invasive assessment in expert centres, with fluid challenge testing during right heart catheterization, which can be useful to unmask post-capillary PH or clarify the contribution of left-heart disease [6, 10].

A structured multidisciplinary team is often lacking in real-world clinical routines, contributing to the ongoing underdiagnosis and undertreatment of PAH among patients with SSc.

The lack of a solid national network for the management of SSc-PAH may be a limitation for peripheral centres, as not all rheumatologists in local hospitals have direct and timely access to PAH referral centres. Referrals are frequently based on personal contacts rather than standardized protocols [11] and regional disparities in drug access may further complicate the uniform application of therapeutic recommendations.

In addition, appropriate care coordination is not implemented and the role of the dedicated case manager or specialized nursing staff caring for PAH is overlooked, although these professionals are essential for facilitating referrals, supporting patients and ensuring continuity in follow-up [6].

### Opportunities and strengths

While expert centres recognize that SSc-PAH is associated with a poor prognosis, the translation of this awareness into consistently proactive and systematic screening practices for SSc patients with a disease duration >3 years remains variable [1, 6].

Validated screening algorithms, such as DETECT, and broader multiparameter approaches recommended by the ESC/ERS guidelines have been developed to facilitate the identification of at-risk patients even in the early SSc stages and in the absence of overt cardiopulmonary symptoms, making them valuable tools for rheumatologists [6, 11, 12]. The DETECT algorithm, endorsed by the ESC/ERS guidelines, is based on a two-step process that relies on non-invasive, widely available clinical data [e.g. forced vital capacity (FVC), N-terminal prohormone of brain natriuretic peptide (NT-proBNP), urate, ECG], each one with a specific numerical weight based on their contribution to PAH risk. When the score is above the threshold, the algorithm recommends proceeding to a first step with echocardiography and then to a second step with right heart catheterization [6, 12]. The DETECT algorithm is easy to apply and its implementation in clinical practice has shown promising results, enabling timely identification of patients who may otherwise be missed by traditional screening methods [11].

Beyond the DETECT algorithm, a clear set of clinical and instrumental markers, such as elevated NT-proBNP levels, reduced performance on the 6-min walk test (6MWD), abnormalities on echocardiography and findings on high-resolution CT (HRCT), are now widely recognized as signs warranting further evaluation [6]. Pulmonary function tests (PFTs), particularly diffusion capacity of carbon monoxide (DLCO) assessment, should be systematically integrated into PAH screening in SSc patients. A DLCO reduction, especially associated with a preserved FVC and an increased FVC:DLCO ratio, may represent an early sign of pulmonary vascular involvement and should require further evaluation. In addition, PFTs and HRCT help in distinguishing PAH from PH associated with interstitial lung disease, supporting a multidisciplinary assessment of SSc patients [6, 13]. Multimodal imaging, including echocardiography, HRCT and, in selected cases, cardiac magnetic resonance, may also help identify mixed PH phenotypes frequently observed in SSc patients, supporting a more accurate phenotypic characterization and multidisciplinary management approach [6].

Nailfold videocapillaroscopy is safe, non-invasive and cost-effective and, in combination with other markers, identifies patients to be prioritized for annual PAH screening or referred to a specialized centre for right heart assessment [14] because severe capillary microangiopathy correlates with PAH development [15].

Echocardiography can help to diagnose heart failure with reduced ejection fraction (HFrEF) and heart failure with preserved ejection fraction (HFpEF) and may detect elevated pulmonary pressures and other features of PH (dilated right atrium and right ventricle, pulmonary artery enlargement, anomalous left ventricle eccentricity index, short right ventricle outflow tract acceleration time), leading to an echocardiographic probability of PH [6]. However, echocardiography alone may underestimate or overestimate pulmonary pressures, particularly in patients with milder forms of PH. Nevertheless, the presence of high-risk features such as tricuspid regurgitation velocity >3.4 m/s or septal flattening should suggest referral to an expert PH centre for comprehensive evaluation and right heart catheterization [6, 16]. Moreover, a stepwise, composite echocardiographic score may discriminate pre- *vs* postcapillary PH and predict pulmonary vascular disease in patients with a left heart condition [17].

Finally, given the complexity and variability of cardiopulmonary haemodynamics in elderly SSc patients or those affected by left heart conditions, the distinction between post- and precapillary PH can be challenging. Diagnostic clues include a complete clinical and echocardiographic evaluation, and the invasive haemodynamic study may eventually include a dynamic evaluation of the pulmonary circulation [10].

It is well known that a thorough assessment requires a close multidisciplinary collaboration [18] as a team of different specialists is essential for optimizing the care of SSc-PAH patients. Given the heterogeneous and multi-organ nature of SSc, the complexity of diagnosis, the need for tailored treatment and the importance of long-term follow-up are of paramount importance [6, 19]. In expert centres, multidisciplinary teams, involving rheumatologists, cardiologists, pulmonologists, radiologists and specialized nursing staff, have demonstrated clear advantages, including structured diagnostic workflows and shared decision-making. In some regions, formalized care pathways and dedicated referral systems have facilitated timely communication between peripheral and expert centres [20, 21]. In centres that do not have all relevant specialties on-site, management can still be achieved through fast-track referral agreements with external specialists, shared electronic health records and scheduled case discussions via virtual platforms. Regional and national networks that support clinical coordination, continuous education and shared learning can help to reduce the fragmentation of care, demonstrating that integration across local centres and standardized follow-up are achievable when supported by structured collaboration [22].

## Therapeutic strategy: risk stratification tools and treatment escalation strategies

The goal in the treatment of SSc-PAH is to achieve early disease control, reach and maintain a low-risk status and ultimately improve long-term survival and quality of life [6]. Defining an optimal treatment strategy for SSc-PAH patients is challenging and depends on appropriate risk stratification, which can be complex in SSc patients because the 6MWD test may be influenced by mobility limitations due to arthritis, proBNP may be elevated due to heart involvement and functional class definition may be difficult due to multi-organ involvement. Routine incorporation of non-invasive parameters [World Health Organization (WHO) functional class, NT-proBNP, 6MWD) alongside careful echocardiographic and haemodynamic reassessment is essential for risk stratification [6]. Use of the ESC/ERS four-strata model (low, low–intermediate, intermediate–high, high risk) or validated tools like REVEAL Lite 2.0 is recommended at diagnosis and during follow-up, although their predictive accuracy in SSc may be reduced by overlapping systemic manifestations [6, 23]. In this setting, low DLCO should be considered an independent marker of poor prognosis [23].

Awareness of the importance of a prompt therapeutic intervention in SSc patients diagnosed with PAH is still uneven, particularly outside of tertiary care settings. Moreover, even when appropriate therapy is started after PAH diagnosis, long-term monitoring and treatment escalation are frequently lacking in SSc patients, leading to suboptimal outcomes. An additional challenge is that the haemodynamic and clinical phenotype of PAH in SSc is not fixed: patients initially classified as group 1 PH (with PAH driven by intrinsic pulmonary vascular disease) may evolve over time toward group 3 PH if progressive interstitial lung disease leads to increasing parenchymal involvement and hypoxic vasoconstriction. Conversely, mixed or borderline haemodynamic profiles may emerge during follow-up as both vascular and fibrotic components evolve [2, 6, 12]. This dynamic nature of SSc-associated PH underscores the necessity of regular reassessment in expert centres to ensure that management strategies remain aligned with the patient’s current pathophysiology.

**Figure 1:**
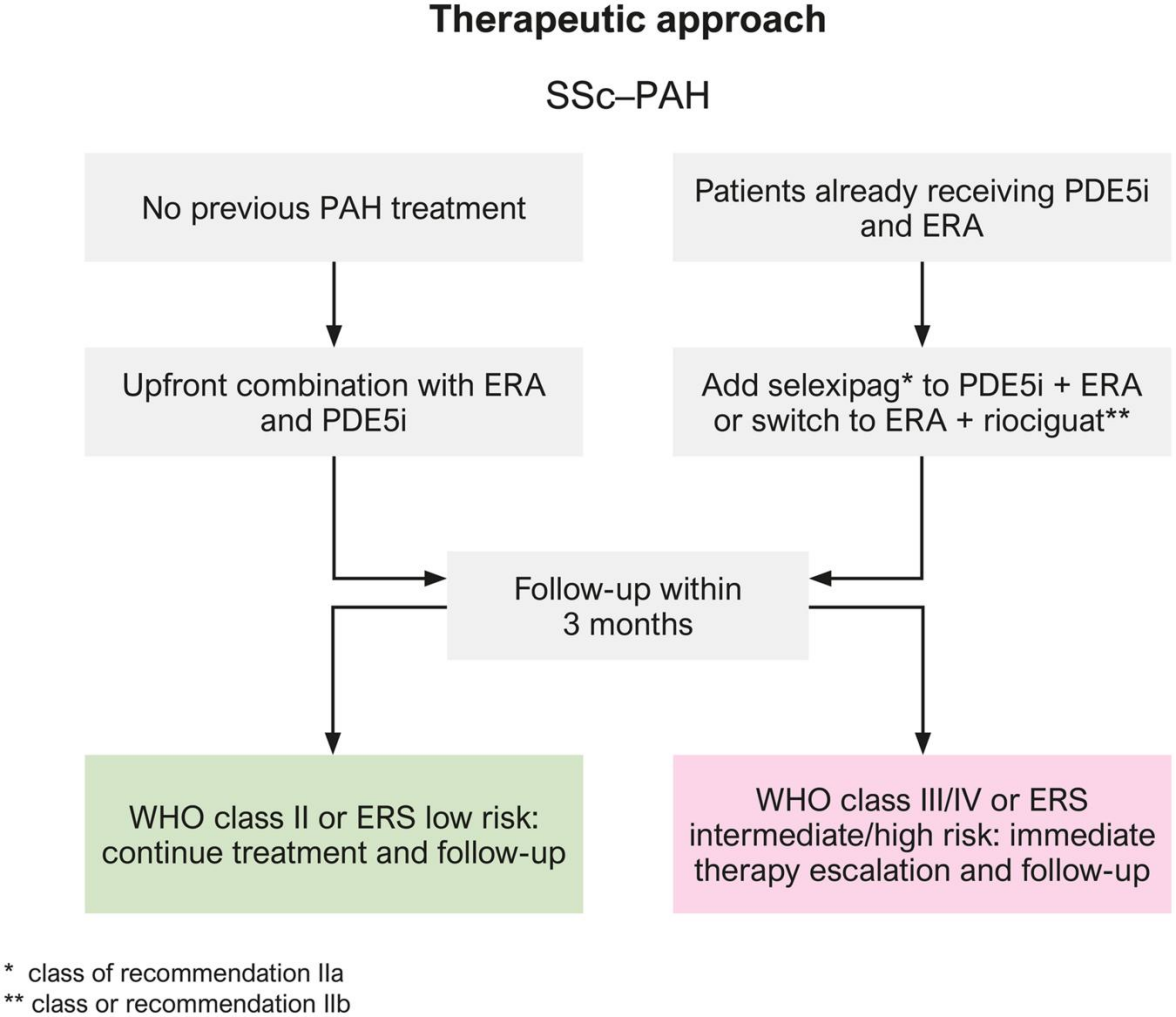
Treatment pathway for SSc-associated PAH

The 2025 EULAR recommendations, according to the ESC/ERS guidelines, advocate for initial combination therapy with an endothelin receptor antagonist (ERA), and a phosphodiesterase type 5 (PDE5) inhibitor is recommended at the time of PAH diagnosis in SSc patients [6, 7]. If a WHO functional class II or ERS-defined low-risk profile is not achieved or maintained, timely escalation to triple therapy including selexipag (class of recommendation IIa) should be considered [7]. In SSc patients already receiving dual therapy with an ERA and a PDE5 inhibitor for the management of digital ulcers, upon PAH diagnosis the rheumatologist should promptly initiate triple combination therapy with the addition of selexipag (class of recommendation IIa) or alternatively a switch from the PDE5 inhibitor to riociguat (class of recommendation IIb) (Fig. 1) [7, 24–26].

The EULAR guidance also emphasizes the centrality of regular risk reassessment in SSc-PAH patients, as PAH progresses more rapidly in these patients [3, 4]. The first follow-up evaluation is crucial and should be performed early after PAH diagnosis, within 3 months, to allow timely therapy escalation. A follow-up at 3–6 months is then recommended, with a multidisciplinary approach involving specialists with experience in SSc-PAH [7]. Indeed, although international guidelines clearly dictate the therapeutic algorithm, their real-world implementation is challenging. Access to advanced therapies is highly heterogeneous due to the organization of regional healthcare. Patients managed in non-specialist centres often lack a multidisciplinary approach and face prolonged bureaucratic approval pathways and restricted access to specific drug classes. This structural limitation could delay the initiation of upfront combination therapy.

Tolerability is another major challenge in daily practice. Initial upfront dual oral combination therapy presents side effects, commonly peripheral oedema, severe headaches, flushing and nasal congestion. In fragile SSc patients suffering from concomitant gastrointestinal involvement or skin ulcers, these side effects can impact compliance, leading to treatment discontinuation. A proactive strategy with close monitoring, gradual drug titration when appropriate and continuous counselling by a dedicated case manager or specialized nurse is essential to maximize adherence. Finally, therapeutic escalation is frequently hindered by clinical and therapeutic inertia. Despite recommendations to use multiparametric risk stratification tools (e.g. REVEAL 2.0 or the ERS/ESC three-strata/four-strata model), many clinicians delay upgrading to triple therapy (including selexipag) or switching to alternative pathways (such as riociguat). This inertia is often driven by a false perception of clinical stability in patients who are ‘not worsening’, ignoring that SSc-PAH progresses rapidly and requires a proactive approach to achieve or maintain a low-risk status. Implementing structured follow-up protocols between rheumatologists and cardiologists is mandatory to overcome these barriers and ensure timely therapeutic adaptation.

Currently, no recommendation can be provided regarding the use of sotatercept, an activin signalling inhibitor, although potential benefit has been reported [27]. Dedicated evidence in SSc-associated PAH is limited and this treatment is not yet addressed in current ESC/ERS guideline recommendations for SSc-PAH management as definitive evidence defining its role within the current therapeutic algorithm is still lacking [6].

Given the complex features of SSc patients, rheumatologists, pulmonologists and cardiologists should be involved in their management throughout treatment and follow-up. Since the clinical phenotype may evolve over time, with the potential emergence of left heart involvement or pulmonary veno-occlusive features, the need for continuous reassessment is central to ensure optimal long-term patient management [28].

## Bridging the gaps: practical strategies to strengthen SSc-PAH care pathways

**Figure 2:**
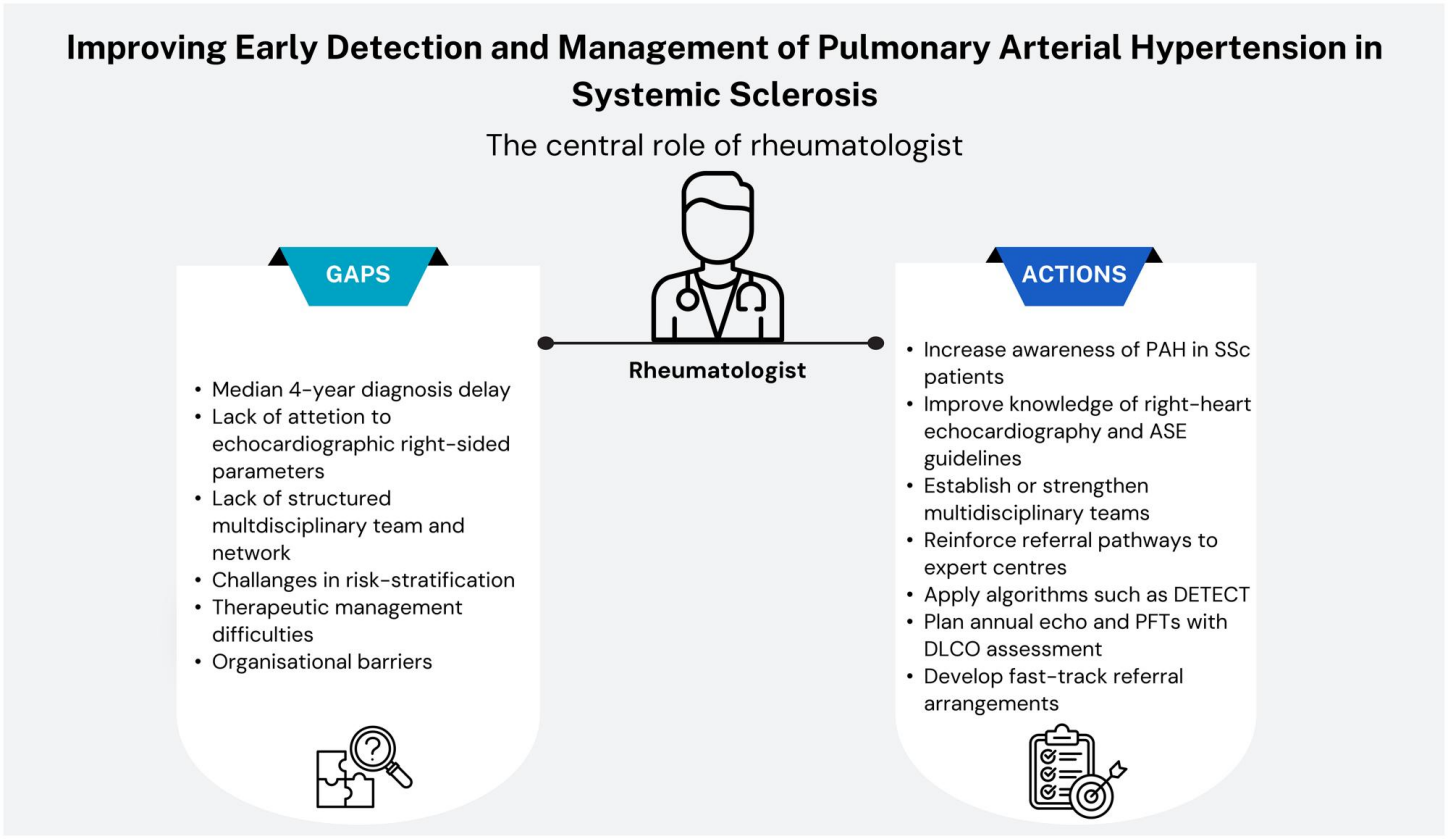
The central role of the rheumatologist in addressing gaps for SSc-associated PAH care. In non-expert centres, a stepwise screening approach integrating clinical suspicion, annual PFTs with DLCO assessment, biomarkers, echocardiography and validated tools such as the DETECT algorithm may facilitate earlier identification of patients requiring referral to expert PH centres. ASE: American Society of Echocardiography

For diagnosis and risk stratification of PAH in SSc patients, reference to ESC/ERS PAH guidelines is essential. Nonetheless, SSc-associated PAH presents distinct features compared with idiopathic PAH and its management should be rheumatologist-led, with cardiology input for diagnostic confirmation and treatment decisions tailored to broader systemic involvement [7]. Routine implementation of the DETECT algorithm in rheumatology practice should be actively encouraged (Fig. 2).

Regular cardiopulmonary evaluation is essential in SSc patients, who should undergo at least one transthoracic echocardiogram per year, irrespective of clinical severity. This approach is feasible in all centres, as annual echocardiography is a planned, non-urgent screening examination that can be scheduled in advance. Echocardiography is the cornerstone of screening, upon which additional assessments, such as NT-proBNP measurement, ECG and evaluation of symptoms or signs suggestive of evolving PAH, should be systematically integrated. Echocardiographic findings should be interpreted within the broader clinical context and integrated with PFTs, biomarkers and rheumatologic features through multidisciplinary evaluation to improve pretest probability assessment and optimize referral pathways to PH expert centres. This structured rheumatologist-led strategy supports early identification of patients transitioning toward an intermediate risk profile and ensures timely referral to specialized centres for confirmatory assessment and management optimisation.

Early and proactive therapy escalation is crucial in SSc patients at diagnosis of PAH. Promoting the use of simplified educational tools, such as checklists, can empower non-specialist rheumatologists to recognize key clinical warning signs and refer patients in a timely manner. Encouraging collaboration between rheumatologists, cardiologists and pulmonologists, adapting communication and education strategies to local needs, including the development of region-specific referral maps and interactive resources, can help increase clinician engagement and reduce variability in care. The role of the case manager still needs to be formalized and supported as a central figure in coordinating care pathways, acting as a bridge between primary care and tertiary expertise, supporting patients and specialists in practical and clinical issues in both diagnostic and therapeutic processes.

## Data Availability

No new data were generated or analysed in support of this research.
